# Rhizobacterial Colonization and Management of Bacterial Speck Pathogen in Tomato by *Pseudomonas* spp.

**DOI:** 10.3390/microorganisms11051103

**Published:** 2023-04-23

**Authors:** Mohsen M. Elsharkawy, Amr A. Khedr, Farid Mehiar, Elsayed M. El-Kady, Khairiah Mubarak Alwutayd, Said I. Behiry

**Affiliations:** 1 Department of Agricultural Botany, Faculty of Agriculture, Kafrelsheikh University, Kafr Elsheikh 33516, Egypt; 2 Department of Biology, College of Science, Princess Nourah bint Abdulrahman University, P.O. Box 84428, Riyadh 11671, Saudi Arabia; 3 Agricultural Botany Department, Faculty of Agriculture (Saba Basha), Alexandria University, Alexandria 21531, Egypt

**Keywords:** *Pseudomonas syringae*, chemotaxis activity, rhizobacteria, tomato, systemic resistance, pathogenesis-related genes

## Abstract

Plants and soil microorganisms interact at every stage of growth. *Pseudomonas* spp. are highly regarded for their ability to increase crop production and protection from diseases. The aim of this study is to understand the mechanisms of the rhizobacterial colonization of tomato roots via chemotaxis assay and the activation of tomato resistance against the pathogenic bacterium, *Pseudomonas syringae* pv. tomato DC3000 (Pst). The capillary assay was used to evaluate the chemotaxis response of PGPRs (plant growth-promoting rhizobacteria). The activities of defense enzymes and the expressions of PR (pathogenesis-related) genes were measured using real-time qPCR. Chemotactic responses to malic and citric acids (the most important root exudates found in different plant species) at low concentrations varied substantially among the rhizobacterial isolates (63 species). Beneficial isolates including *Pseudomonas resinovorans* A5, *P. vranovensis* A30, *P. resinovorans* A28, *P. umsongensis* O26, *P. stutzeri* N42, and *P. putida* T15 reacted well to different concentrations of root exudates. *P. putida* T15 demonstrated the most potent anti-Pst activity. At three and six days after inoculation, the greatest levels of polyphenol oxidase and peroxidase activity were reported in the A5 and T15 groups. In tomato, transcript levels of four PR (pathogenesis-related) genes were elevated by rhizobacterial treatments. PGPR isolates alone or in combination with BABA (β-amino butyric acid) up-regulated the transcriptions of *PR1*, *PR2*, *LOX*, and *PAL* genes. Treatments with N42 and T15 resulted in the greatest improvements in tomato growth and yield traits. In conclusion, the results explain the mechanisms of rhizobacterial colonization for the improved management of Pst. Rhizobacterial isolates play a role in tomato’s resistance to Pst via salicylic acid and jasmonic acid pathways.

## 1. Introduction

Rhizobacteria that enhance plant development and health are called PGPR (plant growth-promoting rhizobacteria) [1,2]. Biocontrol agents, especially PGPR, may act against different pathogens. The activation of plant growth by PGPR occurs through changing plant hormone concentrations, nutrient facilitation by biological N_2_ fixation, inorganic phosphate solubilization, organic phosphate mineralization, and siderophores formation [3,4,5,6]. Antibiosis against phytopathogens, systemic resistance stimulation in plants, and the regulation of the growth of phytopathogens by forming iron-limiting conditions via the production of siderophores are examples of indirect mechanisms that reduce the effect of phytopathogens on plant growth [7]. PGPR have been utilized in commercial applications for disease biocontrol and phytoremediation, such as *Pseudomonas putida* [8]. Biofertilizers, phytostimulators, and biopesticides are the three forms of PGPR, based on their modes of action [9]. To accomplish these beneficial effects, PGPR colonization of the roots is clearly needed [7]. PGPR may be more effective in agricultural production by further understanding the dynamic relationships between plants and microbes. In addition to growth-promoting properties, establishment and persistence in the rhizosphere are essential for an effective bacterium. The activation of various pathways is needed for antagonistic bacteria to exhibit such properties. However, little is known about the potential mechanisms underlying these positive effects through the modification of the rhizosphere microbial community and soil functionality, despite the encouraging plant growth promotion results commonly reported and mostly attributed to phytohormones or other organic compounds produced by PGPR isolates.

Rhizosphere competence is the potential of rhizobacteria to colonize the surface of roots and compete for nutrients released by roots in rhizosphere soil. The three major forms of rhizosphere competence characteristics are chemotaxis, adhesion, and growth. PGPR isolates may have rhizosphere competence based on the transcription of genes associated with chemotaxis and adhesion to surfaces. Characteristics of rhizosphere competence, such as chemotaxis against root exudates (RE), adhesion functions, and RE metabolization, were widely investigated in order to reveal the roles of active rhizosphere colonization [10]. RE perform significant roles in the plant-microbe relationship [11]. RE are a source of carbon for soil microbes. Additionally, they serve as a signal to encourage the growth of microbes [12]. Organic acids such as malic and citric acid are major components of RE [13]. The role of organic acids in controlling plant-microbe interactions was reported in numerous studies [14,15]. For example, *Pseudomonas fluorescens* WCS365 exhibits chemotactic responses to different organic acids [16]. *Bacillus subtilis* FB17 is attracted to the malic acid released by plants’ roots [17]. Different plant species have an impact on the composition of RE. This relationship is thought to have an effect on microbial communities in the rhizosphere soil [18]. Exudates from plant roots develop their own microbial communities in the soil [19]. For example, among low and high-density stands in Montana, the fungal community of knapweed decreased significantly in nature and diversity [20]. Hence, plants use RE to select specific microbial species from a variety of microorganisms in the soil. These compounds were compatible with the bacteria found in the rhizosphere [19]. Colonization and chemotaxis are the two most important components of plant-microbe relationships. Bacterial colonization begins with the chemotaxis of root exudates [21]. For instance, by competing for nutrients and space, *B. amyloliquefaciens* SQR9 protects cucumber from infection with soil-borne pathogens [22]. *B. subtilis* N11, on the other side, can colonize the rhizosphere of banana plants and inhibit pathogen infection [23].

The tomato speck disease pathogen *P. syringae* pv. *tomato* (Pst) DC3000 has gained notoriety as a model organism for investigating the complex relationships between plants and pathogens [24]. Pst is responsible for bacterial speck disease, which is one of the most serious foliar diseases of tomato due to the substantial losses it may cause when the temperature is low and moisture is high [25]. It is still difficult to manage this disease. Although there are tomato genotypes that exhibit some resistance to Pst, the disease has been proven to be able to also overcome this resistance [26,27]. PGPR have been shown to activate systemic resistance to bacteria, fungi, and viruses in different plants. Some PGPR strains that induce ISR (induced systemic resistance) are associated with SAR (systemic acquired resistance) progress [28]. The two signals (SA and JA) coordinately stimulated 50 defense-related genes in *Arabidopsis* plants subjected to different defense-inducing methods [29]. Through a signaling pathway interaction, SA controls the defensive mechanisms in combination with ethylene [30]. Plants may have evolved separate signaling pathways to improve their defensive responses against specific pathogens, depending on their virulence methods [31]. Resistance to (Cucumber mosaic virus) CMV in tobacco plants treated with *Bacillus* spp. was concomitant with a higher transcription of the PR genes (*NPR1* and *Coi1*) [32]. The aims of this research are to examine the chemotactic behavior of different rhizobacterial isolates belonging to *Pseudomonas* spp. through capillary assay to evaluate the effect of the most effective PGPR strains in terms of chemotactic activity on tomato growth and resistance against Pst, and to explore the various mechanisms involved in the resistance induction against Pst using real-time quantitative PCR analysis.

## 2. Materials and Methods

### 2.1. Chemotaxis Activity of PGPRs

#### 2.1.1. Collecting Root Exudates

RE were collected [33]. Tomato seeds (*Lycopersicon esculentum* cv. *Pantelosa*), obtained from the Ministry of Agriculture, Egypt, were sterilized using NaOCl (sodium hypochlorite solution at 2% *v*/*v*, for 2 min) followed by washing three times in SDW (sterile distilled water). In a Petri dish, seeds (100 seeds) were put on Hoagland solution-wetted filter papers in the darkness at 25 °C for germination. Exudates from the roots were gathered in a magnate stirrer with a perforated tray filled with 80 mL of Hoagland solution. Eighty germinated seedlings with their roots in the solution were put on the tray. Exudates were obtained by filtration through Whatman No. 3 filter papers after 1 week of growth at 18 °C to eliminate solid plant contents, and then frozen quickly with liquid nitrogen. On solidified King’s B medium, a sample of exudates was taken directly from the magnate vessels and checked for contamination. The solid material was re-dissolved in 2 mL sterile water after the exudates were lyophilized. These exudates were purified through a 0.45 µm Millipore membrane to eliminate undissolved particles and deposited at −20 °C until needed. Samples were identified and confirmed using liquid chromatography-mass spectrometry (Agilent, Santa Clara, CA, USA) [34].

#### 2.1.2. Chemotactic Response of Bacterial Isolates to Organic Acids in a “Drop” Assay

The “drop” assay was utilized to conduct chemotaxis experiments with slight modifications [35]. After 24 h of incubation on Kings B medium, cells were diluted to 10^−2^ in 150 mL of chemotaxis buffer containing 1% succinic acid. Forty ml of samples were resuspended in 12 mL chemotaxis buffer (100 mM potassium phosphate [pH7.0]/20 mM EDTA) when cells reached the early logarithmic phase (OD_600_ of 0.12). To achieve a viscosity of 4000 cP in a 2% aqueous solution, the cell suspension was treated with an aqueous solution (up to 15 mL) of 1% hydroxypropyl methyl cellulose (Sigma-Aldrich). The resultant cell suspension was moved to a Petri dish (60 mm in diameter), where it produced a 3 mm thick layer. A drop (10 µL) of concentrated (50-fold) root exudates (citric acid (100 mM) and malic acid (100 mM) was placed into the dish’s center at a concentration equal to or less than 0.1 M. After incubation at room temperature for 0.5 to 2 h, the plates were examined for the presence of a clear zone around the bacteria as criteria for a chemotactic reaction. Any turbidity rings that formed in the following 30 min were noted to determine the extent of the chemotactic reaction.

#### 2.1.3. Chemotaxis Assay (Capillary Assay)

A modified capillary test based on Adler’s method was used for the quantitative assessment of isolates’ chemotaxis response to citric acid [36]. Sixty-three PGPR isolates were tested in this experiment. The identification of all strains was established by comparing 16S rDNA sequences to those of the strains. The pathogen *P. syringae* pv. *tomato* DC3000 and rhizobacterial isolates were obtained from Gifu university, Japan. The KB medium was used to grow the strains until they reached the log phase (OD_600_ = 0.12). The cells were centrifuged and cleaned twice using chemotaxis buffer before being resuspended in the same buffer. Six mL of the above-mentioned cell suspension were placed on a Petri dish (60 mm in diameter). Three concentrations (1, 10, and 50 μM) of organic acids (malic acid and citric acid) were put into standard 2 μL capillary tubes and then immersed in the cell suspension. Static incubation at room temperature for 30 min after removing the capillary’s contents into a sterile Eppendorf tube was performed. The suspension was diluted and then plated on KB plates. In KB plates cultured for 24 h at 30 °C, the CFU was measured. Each treatment was carried out three times in total.

### 2.2. Induction Treatments with PGPRs

Tomato seeds were sterilized and put on wetted filter papers in Petri dishes under sterilized conditions for germination at 25 °C in the dark. The vermiculite was packed into sterilized polypropylene bags and autoclaved for 6 min at 121 °C (three times at 12 h intervals). Sterilized pots (25 cm in diam) with autoclaved potting medium were planted with tomato seeds and irrigated three times/week. Two weeks of growing in a growth chamber at 30 °C with a 16 h light/8 h dark photoperiod were used to transplant the germinated seeds. PGPR isolates, attracted to low concentrations of citric acid, were grown in KB medium and shaken at 160 rpm and 30 °C for 24 h. The cells were collected by centrifugation at 12,000 rpm for 10 min, then washed with SDW and resuspended in 10 MgCl_2_. The harvested bacterial suspensions were adjusted to 1 × 10^8^ CFU. Soil was inoculated with different bacterial suspensions. An additional treatment (booster) was conducted 1 day before pathogen inoculation by soil drenching with β-amino butyric acid (BABA). A stock solution of BABA (Syngenta Research, Triangle Park, NC, USA) at 0.33 M was used. *P. syringae* pv. *tomato* DC3000 inoculation was done as described by Elsharkawy et al. [37]. Five days after *Pst* inoculation, the severity of the disease was measured by measuring the area of infected leaves on a scale from 0 (no symptoms) to 100 (severe necrotic symptoms). The number of *Pst* was determined on tomato leaves [37].

### 2.3. Assessment of Defense-Related Enzymes

Enzyme extracts were produced using the approved procedures by Elsharkawy et al. [38]. Tomato leaf samples were collected and pulverized in a mortar and pestle containing liquid nitrogen at three and six days post *Pst* inoculation (DPI). After grinding for 30 s, the powder was homogenized in a mortar and pestle with 3 mL of sodium phosphate buffer pH 6.8 (0.01 M). A refrigerated centrifuge was used to clarify the filtrates for 15 min at 6000 rpm after filtering the triturated tissues through cheesecloth. The clear supernatant was used to measure defense enzymes using a spectrophotometer (DR 5000 UV-VIS-Hyxo).

The activity of peroxidase (POX) in the obtained samples was determined using spectrophotometric analysis. Although there are many different types of POX enzymes, we chose guaiacol as a marker for general POX activity. Phosphate buffer (2.5 mL, pH7.0) and 0.2 mL enzyme extract were added to all test tubes (2 sets). Guaiacol solution (1%, 0.2 mL) was added and stirred in the experimental set. For 15–20 min, both sets were kept at room temperature. Subsequently, the contents of all the test tubes were then carefully mixed with 0.1 mL of 0.3% H_2_O_2_. Distilled water (0.2 mL), 0.1 mL of 0.3 % H_2_O_2_, and 2.5 mL of phosphate buffer (pH 7.0) were used to prepare the blank. A spectrophotometer was used to measure the absorbance at 470 nm. Each measurement was repeated three times.

The enzyme extract was utilized to measure polyphenol oxidase activity (PPO). Using phosphate buffer (pH 7.3), the final volume of the extract was increased to 25 mL, and the filtrate was centrifuged for 1 h at 15,000 rpm. The fraction precipitated between 45 and 95%, and saturation was obtained and re-dissolved in phosphate buffer after an ammonium sulphate fractionation. The mixture was then dialyzed in cellulose dialysis tubing at 4 °C. The activities of catecholase and cresolase were assessed spectrophotometrically [39]. To test the catecholase activity, a 30 mM 4-methyl catechol (4MC) substrate in a sodium acetate buffer (10 mM, pH 4.5) was used. Regarding crude enzyme extract (1 mL), the reaction was initiated by adding 1 mL of the substrate and 3 mL of phosphate buffer (pH 7.3) to the mixture. A spectrophotometer was used to measure the change in absorbance at 400 nm.

### 2.4. Molecular Investigation

The Total RNA Purification Kit (Thermo Scientific, Fermentas (Waltham, MA, USA), #K0731) was used to extract RNA from tomato leaves according to the manufacturer’s procedure. The Reverse Transcription Kit (Thermo Scientific, Fermentas, #EP0451) was used for cDNA synthesis. Template RNA (5 µL) was put to a sterile, nuclease-free tube on ice, followed by 0.5 μL Oligo dT, and the volume was increased to 12.5 μL using DEPC-treated water. On the nuclease-free tube holding the component of the first step, 4 μL of 5× reaction buffer, 0.5 μL of RiboLock RNase Inhibitor, 2 μL of dNTP Mix, and 1 μL of RevertAid^TM^ H Minus Reverse Transcriptase were added. The mixture was gently mixed before being incubated at 42 °C for 60 min. To ensure that the quantities of RNA and cDNA are pure enough for real-time PCR, the concentrations were quantified. The Q5000 (UV-Vis spectrophotometer Q5000/USA) was used to perform all required measurements and computations automatically. The expression of target genes mRNAs was measured using real-time PCR with SYBR Green. The cDNA was amplified using 2× Maxima SYBR Green/ROX qPCR Master Mix (Thermo Scientific, USA, #K0221) and gene specific primers according to the manufacturer’s procedure. Table 1 lists the primers used in the amplification, including pathogenesis-related genes of *PR1*, *PR2*, *lipoxygenase (LOX)*, and *Phenylalanine ammonia lyase (PAL)*. Firstly, the polymerase chain reaction (PCR) mixture was denatured at 94 °C for 5 min, followed by 40 cycles of denaturation for 30 s at 94 °C, annealing for 30 s at 55 °C, and extension for 30 s at 72 °C. The housekeeping gene (*Ubectin 13*) serves as a normalizer for calculating relative gene expression or fold change in the target gene. The 2^−∆∆Ct^ method was employed to equalize the quantities cycle threshold (Ct) of the target gene with the quantities Ct of the housekeeping gene [40]. In both the target and reference genes, the control group served as a calibrator, while the other groups served as test groups.

### 2.5. Plant Growth under Field Experiments

On a loamy soil, 15 treatments were applied in plots of 15 plants for a total of 225 tomato plants on a raised bed at a spacing of 0.55 m. Treatments were *P. umsongensis* O26, *P. vranovensis* A30, *P. resinovorans* A5, *P. resinovorans* A28, *P. resinovorans* A33, *P. resinovorans* A47, *P. brassicacearum* N6, *P. brassicacearum* N32, *P. putida* T15, *P. stutzeri* N42, *P. putida* C21, *P. aeruginosa* B30, *P. alcaligenes* B5, *P. alcaligenes* B16, and control. Tomatoes were planted in rows of 120 × 40 cm, with a row length of 25 m. The experiments used a split-plot design with a randomized complete block and were repeated three times. The plants were irrigated using the drip method. The application of 14 PGPR isolates (1 × 10^8^ CFU/cm^3^) was performed as soil drench at 2 days before pathogen inoculation [2]. Plants in the control group were treated with sterile water. Cumulative yield (kg/plant), plant height (cm), fresh weight (g), dry weight (g), number of branches, chlorophyll contents, number of fruits per plant, average fruit weight (g), leaf area (cm^2^), number of flowers per plant, vitamin C (mg/100 g), titratable acidity, and total soluble solids (TSS, %) were all used to evaluate the growth enhancement by bacterial treatments.

### 2.6. Statistical Analysis

Means were used to represent all data. Fisher’s least significant difference (LSD) test was employed to evaluate statistical significance [38]. The experiments were performed three times. All analyses were performed with a significance level of *p* ≤ 0.05 using XLSTAT PRO (statistical analysis software, Addinsoft 2016.02.270444 version, Paris, France).

## 3. Results

### 3.1. Chemotaxis Activity of PGPRs

The chemotactic response of 63 bacterial isolates to malic and citric acids was investigated to identify the reason for the biocontrol agents’ preferred colonization pattern (Table 2). Isolates including *P. resinovorans* A5, *P. resinovorans* A47, *P. umsongensis* O26, *P. putida* C21, and *P. putida* T15 reacted well to both concentrations of root exudates (Table 2). B5 and B16, on the other hand, may be attracted to malic acid, but not to citric acid (Table 2).

**Figure 1:**
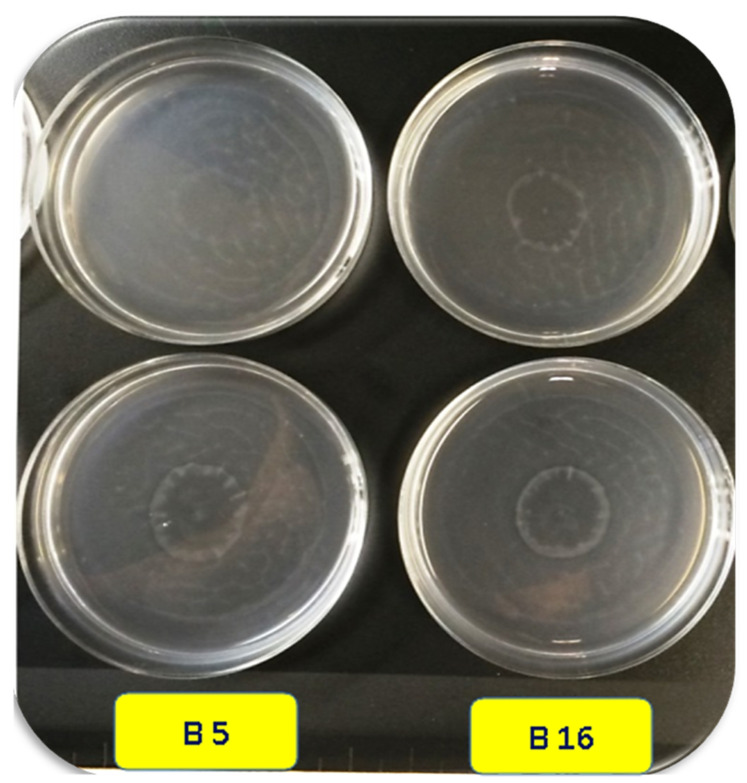
Chemotactic response of the isolates B5 and B16 in response to 10-µL drops of malic and citric acids at concentrations of 100 mM using drop assay.

PGPRs, particularly *P. umsongensis* O26, *P. vranovensis* A30, *P. resinovorans* A5, *P. resinovorans* A28, *P. stutzeri* N42, and *P. putida* T15, responded clearly to chemotaxis buffer concentrated root exudates (malic acid and citric acid), in contrast to the non-treated control (Table 3). B11 and C37 induced an inconspicuous chemotactic response to citric acid. Neither B5 nor B16 elicited a reaction (Figure 1). When examined in 10-µL drops at concentrations of 100 mM, the major amino acids found in tomato root exudate, L-glutamic acid, L-aspartic acid, L-leucine, L-isoleucine, and L-lysine, all elicited a response of PGPR isolates. The threshold concentration of organic acids that may cause chemotaxis was determined by testing various concentrations of organic acids in 10-µL drops. Citric acid and malic acid responses were started at doses as low as 10 to 50 mM (Table 3). The best isolates in the responses to low doses of citric and malic acids were shown compared with the isolates *Pseudomonas alcaligenes* B5 and *Pseudomonas alcaligenes* B16 (Table 3).

**Figure 2:**
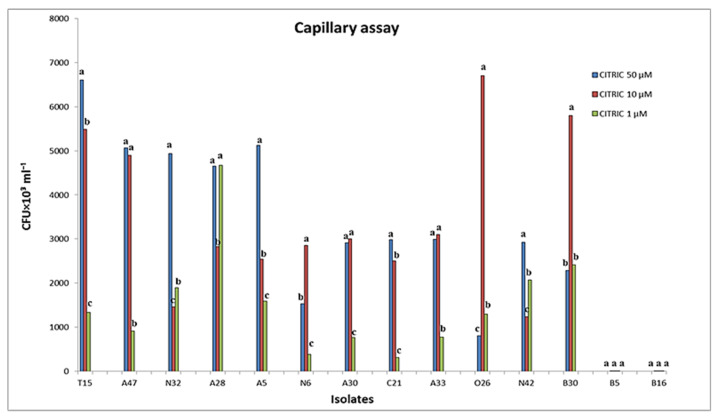
Chemotactic response of PGPRs towards different concentrations of malic and citric acids evaluated by capillary assay. Columns with different letters are statistically different according to the Fisher’s LSD test (*p* ≤ 0.05).

The chemotactic response of PGPRs to different doses of malic and citric acids was determined using a modified quantitative capillary chemotaxis test. Citric acid, found in tomato root exudates, was shown to attract *P. umsongensis* O26, *P. vranovensis* A30, *P. resinovorans* A5, *P. resinovorans* A28, *P. stutzeri* N42, and *P. putida* T15 at concentrations ranging from 1 to 50 mM (Figure 2). Malic and citric acids (1, 10, and 50 mM) showed clear positive and concentration-dependent chemotactic behavior in PGPR strains, with the most effective concentration ranging from 10 to 50 mM (Figure 2). Only B5 and B16 chemotactic responses were induced by malic acid at concentrations of 1, 10, and 50 mM, without any response toward all concentrations of citric acids (Figure 2).

### 3.2. Induction of Systemic Resistance against Pst by PGPR

**Figure 3:**
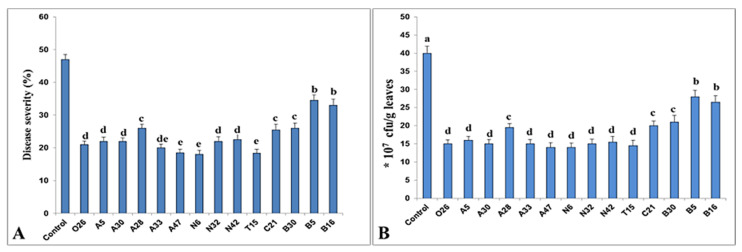
Induced suppression of disease symptoms (**A**) and number of *Pst* bacteria (**B**) in tomato plants in response to root treatment with PGPRs. Columns with different letters are statistically different according to the Fisher’s LSD test (*p* ≤ 0.05).

Root treatment with PGPR resulted in induced systemic resistance (ISR) in tomato against *Pst*. The defensive mechanisms mediated by PGPR were described using a tomato-based model system. A screening procedure using root treatment was done to explore the most effective PGPR strains in terms of chemotactic activity and, consequently, the most effective isolates against *Pst*. ISR against the pathogen was measured five days after a challenge by calculating disease severity as a percentage, and *Pst* proliferation was checked in the leaves. T15 had the most remarkable outcomes in terms of lowering disease severity (18.3%) and pathogen proliferation (14.5) (Figure 3).

**Figure 4:**
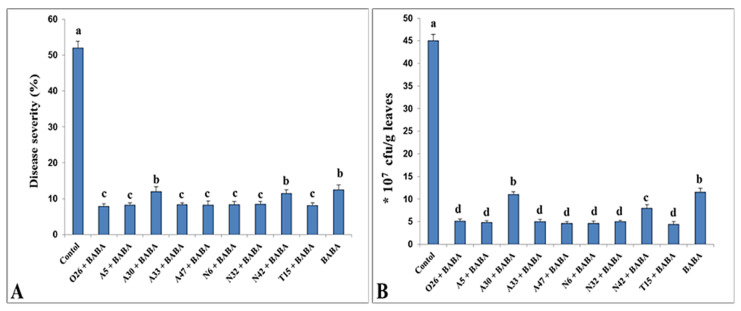
Induced suppression of disease symptoms (**A**) and number of *Pst* bacteria (**B**) in tomato plants in response to dual treatments with PGPR and BABA. Columns with different letters are statistically different according to the Fisher’s LSD test (*p* ≤ 0.05).

Among 14 PGPR isolates, the most efficient nine isolates were selected and used as dual treatments with BABA against *Pst*. Results showed that the treatments exhibited substantial suppression of disease symptoms without any symptoms of infection (Figure 4). According to these findings, root dual treatments with BABA and *P. umsongensis* O26, *P. resinovorans* A5, *P. resinovorans* A33, *P. resinovorans* A47, *P. brassicacearum* N6, *P. brassicacearum* N32, and *P. putida* T15 were more resistant to *Pst*-caused foliar disease, and showed a considerable decrease in disease severity (7.8, 8.2, 8.4, 8.2, 8.3, 8.5, and 8.1%, respectively) and the proliferation of *Pst* in the leaves (5.1, 4.8, 5, 4.6, 4.6, 5, and 4.4, respectively) compared with control plants (52 and 45%, respectively).

### 3.3. Effects of PGPR Treatments on Enzyme Activities in Plants Infected with Pst

Peroxidase and polyphenol oxidase activities at 3 and 6 days post-inoculation (DPI) in different PGPR treatments were presented in Table 4. PGPR isolates induced considerable increases in POX and PPO activity in tomato plants compared with the control treatment (Table 4). Additionally, it is clear from the data that POX and PPO activities were higher at 3 DPI than 6 DPI. PGPR isolates; *P. resinovorans* A5, *P. resinovorans* A33, *P. resinovorans* A47, *P. stutzeri* N42, *P. putida* T15, and *P. putida* C21 recorded the highest values of POX and PPO activities at 3 and 6 DPI (Table 4).

PPO and POX activities were determined after 3 and 6 DPI in plants treated with both PGPR isolates and BABA or BABA alone (Table 5). The obtained data showed that the most effective treatment was the dual treatment with T15 and BABA, which recorded 66.0 and 49.4 for POX at 3 and 6 DPI, and 45.4 and 41.1 for PPO at 3 and 6 DPI, respectively, and consequently caused higher reduction values of bacterial leaf speck disease than the untreated inoculated control (Table 5).

### 3.4. Effects of PGPR Treatments on Plant Growth and Yield

The results revealed that bacterial treatments substantially impacted fruit production, vitamin C, and the number of fruits per plant (Table 6 and Table 7). A significant increase in the yield was achieved using A5 (5.8 kg/plant), A30 (5.7 kg/plant), A47 (5.9 kg/plant), N6 (5.9 kg/plant), T15 (6.2 kg/plant) and C21 (5.9 kg/plant) as compared with the control (3.3 kg/plant) (Table 6). PGPR-treated plants outperformed their untreated controls in terms of yield measures. Treatment with T15 exhibited the highest increase in the number of flowers and fruits and weight of fruits/plant compared with other isolates and control treatment. Additionally, the isolates *P. resinovorans* A5, *P. resinovorans* A47, *P. brassicacearum* N6, and *P. putida* C21 showed a higher number of fruits, and fruits weight/plant T15 had the most significant outcomes in terms of fresh and dry weights, plant height, number of branches, leaf area, and chlorophyll content, although there were substantial variations between treatments.

TSS, acidity, and vitamin C concentration were the fruit quality parameters examined. T15 treatment provided the highest vitamin C (137.3 mg/100 g) compared with the control treatment (120.2 mg/100 g). *P. putida* T15, *P. stutzeri* N42, *P. vranovensis* A30, *P. resinovorans* A5, and *P. putida* C21 had the same TSS levels (5.21, 5.19, 5.19, 5.22, and 5.18, respectively), with no statistically significant differences. All treatments were significantly higher than the control. Similar to vitamin C contents, A5, A30, A47, N6, N42, T15, and C21 treatments increased the acidity of tomato fruits (0.58, 0.57, 0.58, 0.58, 0.59, 0.58, and 0.57) compared to the control (0.47), respectively (Table 7).

### 3.5. Molecular Analysis of Defense Genes by Real-Time PCR

After treatment with PGPR isolates alone or in combination with BABA, real-time PCR was performed to assess the relative expression of four pathogenesis-related proteins encoded by the genes *PR1* and *PR2*, *LOX*, and *PAL*. An evaluation of these genes’ transcription levels in the treated tomato and the control was carried out. Nanodrop was used to evaluate the quality and concentration of total RNA, which revealed pure RNA with much higher quantities of RNA (ranging from 950 to 2050 ng/µL). The housekeeping gene encoding the elongation factor *Ubectin 13* was utilized as an internal reference for normalization throughout the real-time PCR experiment, and data were represented as mean ± SEM.

**Figure 5:**
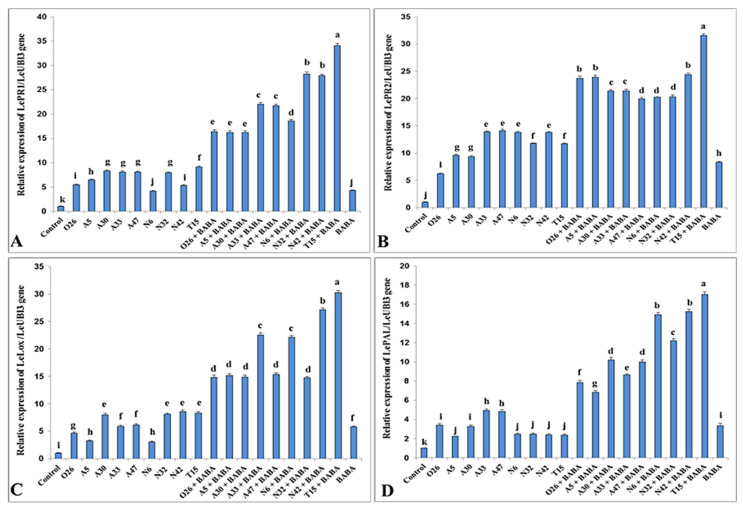
Real-time quantitative PCR analysis of the expression of *PR1* (**A**), *PR2* (**B**), *LOX* (**C**), and *PAL* (**D**) genes in tomato in control group, PGPR treated group, dual treatment with PGPR, and BABA and BABA alone treatment. Columns with different letters are statistically different according to the Fisher’s LSD test (*p* ≤ 0.05).

Compared to control tomato plants, PGPR-treated tomato plants had a substantial increase in *PR1* gene expression before *Pst* inoculation (Figure 5). Plants treated with T15 + BABA showed the highest expression (34.06 folds). The amount of *PR2* gene expression in PGPR-treated tomato plants was substantially higher than in control plants (Figure 5). In addition, dual treatment with T15 and BABA resulted in the highest up-regulation of *PR2* gene expression (31.56 folds), followed by dual treatments with N42 + BABA, A5 + BABA and O26 + BABA.

The mean value of *PR2* gene expression in PGPR-treated tomato plants was substantially higher than in the control plants (Figure 5). Gene expression was not significantly different between O26 + BABA, A5 + BABA and A30 + BABA treatments. When comparing all treatments, dual treatment with T15 and BABA showed the highest expression of the *PR1* gene than other treatments. *PAL* gene expression was significantly increased in plants treated with PGPR compared to the control (Figure 5). T15 + BABA, N42 + BABA, and N6 + BABA were significantly more elevated than the other treatments in all groups. Furthermore, plants treated with T15 + BABA had a greater up-regulation of *PAL* gene expression than plants treated with N42 + BABA or N6 + BABA (Figure 5).

## 4. Discussion

We examined the chemo-attractants in 63 root-colonizing rhizobacterial strains and evaluated their colonizing and chemotactic behavior. The movement of an organism in reaction to a chemical stimulation is known as chemotaxis. This is crucial because bacteria move toward the areas with the highest concentrations of food molecules (such as glucose). Carbon-based chemicals make up the majority of root exudates [41]. Soil microbes need organic acids which may serve as food and signal molecules [14]. The effects of malic and citric acids on the chemotactic response of PGPR were elucidated. The compositions of plant root exudates are closely linked to the chemotaxis response and colonization behavior of bacterial strains. This study offers valuable information with implications for co-evolution between plants and microorganisms in the same niche, as well as improved PGPR-strain selection for agricultural production applications. *P. putida* T15 has the genetic components (chemotaxis, adhesion structures, and antimicrobial activities) to colonize the rhizosphere effectively. Various concentrations of malic and citric acids were examined to establish the chemotaxis threshold concentration. Low quantities of malic and citric acids, such as 10 mM, elicited the response of PGPRs. Exudates stimulated chemotaxis, proliferation, and biofilm formation, which impacted *B. amyloliquefaciens* BNM339 colonization of soybean seeds [42]. The higher favorable reactivity of *P. putida* T15 to tomato root exudates may essentially explain its colonization behavior. Many different organic acids, such as citric and malic acids, are released simultaneously by the bacteria in the rhizosphere at the same time and have been associated with their function in phosphate solubilization.

Fourteen distinctly different PGPR isolates were utilized against *Pst,* and the protective effect and mechanism of the PGPR in tomato plants were elucidated. Our findings show that only PGPR treatment is sufficient to protect plants. In tomato, a mixture of PGPR and BABA substantially increased resistance to *Pst*. The number of symptomatic leaves, the proliferation of *Pst* in the leaves, and the severity of the disease were all decreased as a result of PGPR treatment. In tomato plants, the function of defensive signaling molecules has been investigated using real-time PCR. The fact that PGPR alone or in combination with BABA substantially upregulated the pathogenesis-related genes (*PR1*, *PR2*, *LOX*, and *PAL*) explains their importance in systemic resistance to *Pst*. ISR refers to the reduction in the severity or incidence of disease caused by PGPR-elicited host defenses that are spatially separated from pathogens [43]. The stimulation of PGPR in plants leads to indirect systemic resistance to pathogens [44,45]. In *Arabidopsis* and tobacco, the systemic resistance generated by the bacteria *Serratia marcescens* 90–166 or *Pseudomonas fluorescens* WCS417 is independent of SA accumulation [46]. *P. aeruginosa* 7NSK2, on the other hand, used the SA-dependent pathway to elicit ISR against *Botrytis cinerea* in tomato and *Tobacco mosaic virus* in tobacco [47]. Using *Arabidopsis* mutant lines, a model pathway for signal transduction in PGPR-mediated ISR was established [2]. ISR induced by PGPR is dependent on jasmonic acid (JA), ethylene, and SA, according to the proposed pathway [2]. The treatments *P. putida* T15 and *P. putida* T15 + BABA substantially reduced the severity of *Pst* (*p* ≤ 0.05). When compared to the control, PGPR isolates substantially enhanced all the tested plant growth parameters. Individual *P. putida* T15 treatment exhibited the most substantial increases in tomato growth and yield compared to other treatments. PGPR isolates outperformed the control in all plant growth and morphological parameters. PGPR has been shown to improve plant growth via a number of methods, including mineral phosphate solubilization, biological nitrogen fixation, plant hormone production, and stress reduction [5]. PGPR may also have an indirect impact on plant development by keeping pathogens from deleterious effects.

In conclusion, the processes by which the plant recognizes helpful microorganisms were addressed. Root exudates (malic and citric acids) attract beneficial rhizobacteria leading to the stimulation of defense response in tomato against *Pst*. These results are important for improving management strategies against plant pathogens.

## Figures and Tables

**Table 1:** Forward and reverse primers sequence for pathogenesis-related genes.

Gene	Forward Primer	Reverse Primer	Size	Accession Number
*LePR1*	GCCAAGCTATAACTACGCTACCAAC	GCAAGAAATGAACCACCATCC	139	DQ159948
*LePR2*	GGACACCCTTCCGCTACTCTT	TGTTCCTGCCCCTCCTTTC	81	M80604
*LeLOX*	ATCTCCCAAGTGAAACACCACA	TCATAAACCCTGTCCCATTCTTC	109	U13681
*LePAL*	CTGGGGAAGCTTTTCAGAATC	TGCTGCAAGTTACAAATCCAGAG	150	AW035278
*LeUBI3*	TCCATCTCGTGCTCCGTCT	GAACCTTTCCAGTGTCATCAACC	144	X58253

**Table 2:** Chemotactic response of PGPRs in drop assay in response to malic and citric acid.

Strain	Genus Species	Host Plant	Chemotactic Activity	
			Malic Acid	Citric Acid
N2	*Pseudomonas brassicacearum*	Chinese chive	++	++
N6	*Pseudomonas brassicacearum*	Chinese chive	++	+++
N12	*Pseudomonas brassicacearum*	Chinese chive	++	++
N16	*Pseudomonas brassicacearum*	Chinese chive	+++	+++
N22	*Pseudomonas brassicacearum*	Chinese chive	++	+
N27	*Pseudomonas brassicacearum*	Chinese chive	++	+++
N32	*Pseudomonas brassicacearum*	Chinese chive	+++	+++
N37	*Pseudomonas delhiensis*	Chinese chive	++	++
N39	*Pseudomonas fluorescens*	Chinese chive	++	++
N42	*Pseudomonas stutzeri*	Chinese chive	++	+++
N44	*Pseudomonas plecoglossicida*	Chinese chive	++	++
O10	*Pseudomonas vranovensis*	Onion	++	++
O26	*Pseudomonas umsongensis*	Onion	+++	+++
O29	*Pseudomonas vranovensis*	Onion	++	++
O35	*Burkholderia cepacia*	Onion	+++	+++
O39	*Pseudomonas vranovensis*	Onion	++	++
O41	*Pseudomonas fulval*	Onion	++	++
A5	*Pseudomonas resinovorans*	Garlic	+++	+++
A6	*Pseudomonas vranovensis*	Garlic	++	++
A25	*Pseudomonas vranovensis*	Garlic	+++	+++
A27	*Pseudomonas resinovorans*	Garlic	+++	++
A28	*Pseudomonas resinovorans*	Garlic	+++	+++
A30	*Pseudomonas vranovensis*	Garlic	++	+++
A31	*Pseudomonas resinovorans*	Garlic	++	++
A33	*Pseudomonas resinovorans*	Garlic	+++	+++
A47	*Pseudomonas resinovorans*	Garlic	+++	+++
C1	*Pseudomonas putida*	Cucumber	++	++
C7	*Pseudomonas putida*	Cucumber	+	++
C14	*Pseudomonas putida*	Cucumber	+++	++
C21	*Pseudomonas putida*	Cucumber	+++	+++
C27	*Pseudomonas putida*	Cucumber	+++	+++
C33	*Pseudomonas putida*	Cucumber	++	++
C37	*Pseudomonas* sp.	Cucumber	+++	+++
C40	*Pseudomonas putida*	Cucumber	++	++
C46	*Pseudomonas putida*	Cucumber	+++	++
T1	*Pseudomonas brassicacearum*	Tomato	++	+
T5	*Pseudomonas putida*	Tomato	+	+
T11	*Pseudomonas putida*	Tomato	++	+
T15	*Pseudomonas putida*	Tomato	+++	+++
T21	*Pseudomonas fluorescens*	Tomato	++	+
T26	*Pseudomonas brassicacearum*	Tomato	+++	++
T28	*Pseudomonas alcaligenes*	Tomato	++	++
T31	*Pseudomonas putida*	Tomato	++	++
T36	*Pseudomonas putida*	Tomato	+++	++
T41	*Pseudomonas putida*	Tomato	++	++
B1	*Pseudomonas resinovorans*	Bulk soil	+++	+++
B5	*Pseudomonas alcaligenes*	Bulk soil	+++	−
B6	*Pseudomonas pseudoalcaligenes*	Bulk soil	+	+
B11	*Pseudomonas citronellolis*	Bulk soil	+++	+++
B12	*Pseudomonas resinovorans*	Bulk soil	+++	++
B13	*Pseudomonas resinovorans*	Bulk soil	+++	++
B15	*Pseudomonas resinovorans*	Bulk soil	++	++
B16	*Pseudomonas alcaligenes*	Bulk soil	+++	−
B19	*Pseudomonas resinovorans*	Bulk soil	+	++
B22	*Pseudomonas resinovorans*	Bulk soil	+	+
B28	*Pseudomonas resinovorans*	Bulk soil	+++	++
B29	*pseudomonas resinovorans*	Bulk soil	+++	++
B30	*Pseudomonas aeruginosa*	Bulk soil	+++	+++
B32	*Pseudomonas resinovorans*	Bulk soil	++	++
B36	*Pseudomonas resinovorans*	Bulk soil	+++	++
B39	*Pseudomonas resinovorans*	Bulk soil	++	++
B42	*Pseudomonas alcaligenes*	Bulk soil	++	+
B43	*Pseudomonas panipatensis*	Bulk soil	++	++

**Table 3:** Chemotactic response of PGPRs in response to low concentrations of malic and citric acid.

Strain	Genus Species	Host Plant	Chemotactic Activity					
			Malic Acid mM			Citric Acid mM		
			1	10	50	1	10	50
N6	*Pseudomonas brassicacearum*	Chinese chive	+++	+++	++	+++	++	++
N16	*Pseudomonas brassicacearum*	Chinese chive	+	++	++	+	++	++
N32	*Pseudomonas brassicacearum*	Chinese chive	+++	++	++	+++	+++	++
N42	*Pseudomonas stutzeri*	Chinese chive	+++	+++	+++	+++	+++	++
O26	*Pseudomonas umsongensis*	Onion	+++	+++	++	+++	+++	++
O35	*Bacillus cepacia*	Onion	+++	+++	++	+++	+++	++
A5	*Pseudomonas resinovorans*	Garlic	+++	+++	+++	+++	+++	++
A28	*Pseudomonas resinovorans*	Garlic	+++	+++	++	+++	+++	++
A30	*Pseudomonas vranovensis*	Garlic	+++	+++	++	+++	+++	++
A33	*Pseudomonas resinovorans*	Garlic	++	++	++	+++	++	+
A47	*Pseudomonas resinovorans*	Garlic	+++	+++	++	+++	+++	++
C21	*Pseudomonas putida*	Cucumber	+++	+++	++	+++	++	++
C27	*Pseudomonas putida*	Cucumber	+++	+++	++	+++	+++	++
C37	*Pseudomonas sp.*	Cucumber	++	++	++	++	++	++
T15	*Pseudomonas putida*	Tomato	+++	+++	++	+++	+++	++
B11	*Pseudomonas citronellolis*	Bulk soil	++	++	++	++	++	++
B30	*Pseudomonas aeruginosa*	Bulk soil	+++	+++	+++	+++	+++	++
B5	*Pseudomonas alcaligenes*	Bulk soil	+++	+++	++	−	−	−
B16	*Pseudomonas alcaligenes*	Bulk soil	+++	+++	++	−	−	−

**Table 4:** Activity of peroxidase and polyphenol oxidase enzymes (mg/mL^−1^) on tomato leaves that were treated by PGPRs.

Treatments	Peroxidase		Polyphenol Oxidase	
	3DPI	6DPI	3DPI	6DPI
O26	50.9b	38.9a	25.3b	22.4b
A5	54.8a	39.5a	29.7a	25.2a
A30	51.2b	35.6b	24.9b	21.9b
A28	46.3c	31.5c	20.8c	17.4c
A33	56.1a	41.1a	30.2a	25.4a
A47	55.3a	40.5a	29.9a	24.8a
N6	51.5b	35.9b	25.3b	21.8b
N32	51.3b	36.1b	25.4b	22.2b
N42	55.1a	39.8a	30.5a	25.6a
T15	55.9a	40.7a	30.1a	25.3a
C21	45.9c	30.4c	19.9c	15.7d
B30	46.1c	31.2c	20.6c	15.4d
B5	40.4d	26.3d	19.8c	14.9d
B16	41.9d	25.9d	20.3c	15.3d
Control	19.1e	18.6e	8.9d	8.9e

Different letters indicate significant difference to the Fisher’s LSD test (*p* ≤ 0.05).

**Table 5:** Activity of peroxidase and polyphenol oxidase enzymes (mg/mL^−1^) on tomato leaves that were treated by PGPRs and BABA.

Treatments	Peroxidase		Polyphenol Oxidase	
	3DPI	6DPI	3DPI	6DPI
O26 + BABA	60.6b*	44.6b	35.3c	32.7c
A5 + BABA	66.7a	45.3b	39.9b	32.5c
A30 + BABA	59.1b	39.8c	34.8c	29.4d
A33 + BABA	65.7a	45.6b	35.1c	30.2d
A47 + BABA	65.9a	45.1b	41.2a	36.3b
N6 + BABA	60.4b	41.1c	34.3c	30.8d
N32 + BABA	67.3a	49.2a	41.5a	37.9b
N42 + BABA	66.1a	40.9c	34.3c	30.5d
T15 + BABA	66.0a	49.4a	45.4a	41.1a
BABA	54.2c	33.2d	31.5e	27.9e
Control	21.3d	17.2e	9.5f	9.1f

* Different letters indicate significant difference to the Fisher’s LSD test (*p* ≤ 0.05).

**Table 6:** Effects of PGPRs on tomato growth and yield under field conditions.

Treatments	Morphological and Physiological Characters								
	Plant Height (cm)	Leaf Area (cm)	No. of Branches	Fresh Weight (g)	Dry Weight (g)	Chlorophyll Contents	No. of Flowers	No. of Fruits	Weight of Fruits/Plant
O26	75.0e*	225.4d	20.0c	417.3d	65.5c	44.7b	105.2b	34.6a	4.6b
A5	92.1b	277.8b	24.2b	449.0b	76.3a	48.3a	116.9a	42.8a	5.8a
A30	91.3b	273.5c	23.9b	447.9b	76.2a	47.6a	113.9	38.5b	5.7a
A28	79.4c	226.4d	20.4c	427.5c	68.6b	45.1b	105.8b	35.1c	4.7b
A33	80.2c	226.7d	21.1c	428.2c	69.2b	45.3b	104.1b	35.7c	4.9b
A47	92.3b	278.1b	24.1b	449.2b	77.1a	48.3a	117. 3a	42.9a	5.9a
N6	91.9b	276.9b	23.4b	448.7b	76.9a	48.1a	117.8a	42.6a	5.9a
N32	62.7f	192.4e	16.5d	337.9e	52.8b	39.1c	99.3c	23.2b	4.0c
N42	96.5a	281.7a	26.2a	452.1a	78.3a	48.7a	117.4a	43.0a	6.0a
T15	97.6a	281.9a	25.9a	451.9a	78.1a	49.1a	118.1a	43.5a	6.2a
C21	92.3b	277.3a	25.2a	451.7a	76.9a	47.5a	117.9a	42.6a	5.9a
B30	78.4d	226.7d	20.6c	428.3c	68.5b	45.2b	104.8b	34.8c	4.6b
B5	63.7f	193.1e	17.2d	339.0e	53.3d	38.6c	97.9c	29.7d	4.1c
B16	62.9f	194.2e	17.0d	338.4e	52.9d	39.1c	98.2c	29.9d	4.2c
Control	49.5g	119.2f	13.5e	244.0f	38.1e	35.5d	78.4d	20.6e	3.3d

* Different letters indicate significant difference to the Fisher’s LSD test (*p* ≤ 0.05).

**Table 7:** Effects of PGPRs on quality of tomato fruits.

Treatments	Data Analysis		
	Acidity	Total Soluble Solids (TSS)%	Vitamin C mg/100 g
O26	0.53b*	4.76b	13.14b
A5	0.58a	5.22a	13.54ab
A30	0.57a	5.19a	13.49ab
A28	0.54b	4.69b	13.19b
A33	0.52b	4.75b	13.09b
A47	0.58a	4.78b	13.63a
N6	0.58a	4.73b	13.72a
N32	0.49c	4.81b	12.63c
N42	0.59a	5.19a	13.69a
T15	0.58a	5.21a	13.73a
C21	0.57a	5.18a	13.62a
B30	0.53b	4.79b	13.17b
B5	0.48c	4.75b	12.59c
B16	0.46c	4.73b	12.68c
Control	0.47c	4.69b	12.02d

* Different letters indicate significant difference to the Fisher’s LSD test (*p* ≤ 0.05).

## Data Availability

Not applicable.
